# GTC: A web server for integrating systems biology data with web tools and desktop applications

**DOI:** 10.1186/1751-0473-5-7

**Published:** 2010-07-13

**Authors:** Dan Tenenbaum, J Christopher Bare, Nitin S Baliga

**Affiliations:** 1 Institute for Systems Biology, 1441 N. 34th St., Seattle, WA USA; 2 Departments of Microbiology, and Molecular and Cellular Biology, University of Washington, Seattle, WA, USA

## Abstract

Gaggle Tool Creator (GTC) is a web application which provides access to public annotation, interaction, orthology, and genomic data for hundreds of organisms, and enables instant analysis of the data using many popular web-based and desktop applications.

## Background

There are hundreds of public databases for systems biology data, and an equal number of applications for working with that data. However, it is often difficult to work with data of interest in the desired applications. Databases may not offer programmatic access, or require special scripting. Software tools may imprison the data in such a way that it can only be analyzed by a particular application. Data available for one organism may not be available for another. Individuals may have to download their own copies of databases in order to work with them in nonstandard ways, forcing them into the role of curator. Software tools may not allow users to work with their own data. Applications may only accept data in arcane formats, requiring special conversion.

**Figure 1:**
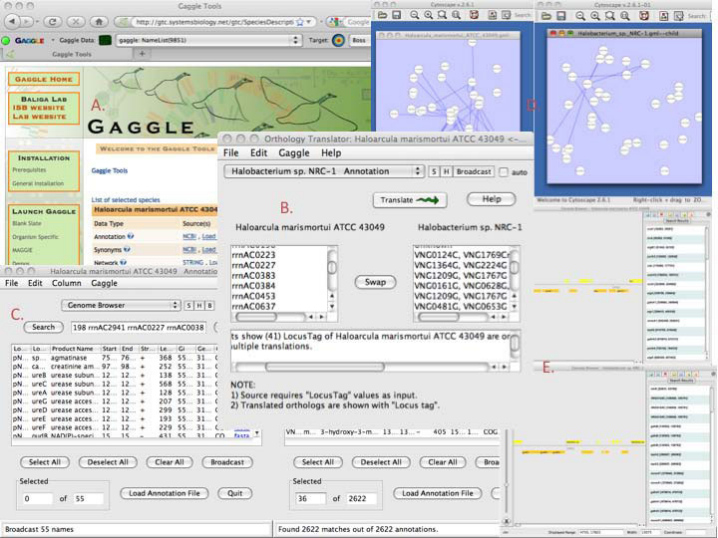
**Exploring the arginine fermentation pathway in *Haloarcula marismortui *and *Halobacterium sp. NRC-1***. Start by opening GTC (A) and selecting these two organisms, which provides the link to launch all the other tools in this figure. A list of genes from *H. marismortui *involved in the arginine fermentation pathway [10] is broadcast to several tools (C, D, E) with one click, including an orthology translator (B), allowing comparable regions in the two organisms to be visualized side by side.

Here we describe Gaggle Tool Creator (GTC), a web application which addresses these problems by providing public data for hundreds of organisms, and making it instantly accessible to many popular, unrelated web resources and desktop applications. This in turn allows sophisticated analyses and novel discoveries to be achieved with just a few mouse clicks (Figure 1).

## Implementation

**Figure 2:**
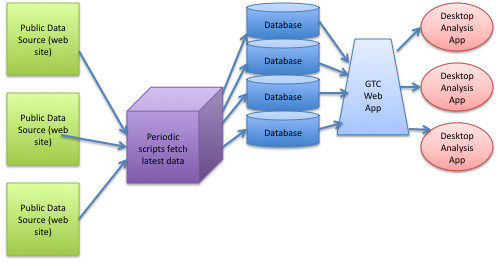
**Flow of GTC**. Periodically run scripts download data from public sources and update local databases. GTC web app enables launching of desktop analysis applications (using Java Web Start) which are loaded with organism-specific data.

GTC is composed of a number of MySQL databases, regularly updated by scheduled scripts which download systems biology data from public sources. These sources, and the method used to download data from each, are as follows: NCBI (web services), STRING[1] (flat file download), BioNetBuilder[2] (flat file download), KEGG[3] (web services), and the UCSC genome browser[4] (flat file download). We currently have data for 500 organisms, with the eventual goal of having data for all sequenced organisms. The core of GTC is a Java web application which makes available links to several applications suited to the analysis of particular types of data. These applications are launched using Java Web Start, a technology which seamlessly pushes software updates to the user's computer. All of these applications implement the Gaggle framework [5] for sharing data between applications and web sites (Figure 2).

## Results and Discussion

GTC's user interface allows the user to choose up to ten organisms to work with.

GTC displays several links for each organism, and the following list describes in greater detail what happens when the user clicks these links.

### Annotation

A desktop application displaying annotation data from NCBI.

### Synonyms

An application that can translate between various naming schemes for genes (Locus Tag, Gene Name, Product Name). Data come from either NCBI or BioNetBuilder.

### Network

The Cytoscape network viewer [6], with data from either STRING or BioNetBuilder.

### GenomeMap

A genome browser, preloaded with genomic data from the UCSC genome web service.

### Orthology Translator

Finds orthologs between any two organisms (invoked when the user chooses exactly two organisms to work with).

Each of these links is paired with another called "Load Your Own" which allows the user to provide their own data. Because Gaggle is a message-passing framework, the user is not limited to the applications listed above and in fact can use any Gaggle-enabled tool; and, using Gaggle's Firegoose extension [7] for the Firefox browser, web sites such as KEGG, STRING, EntrezGene, EntrezProtein, and DAVID [8][9]. (GTC will work with any modern web browser but Firegoose enables two-way communication with Gaggle-enabled web sites.)

## Availability and Requirements

**Project name: **GTC (Gaggle Tool Creator)

**Project home page: **http://gaggle.systemsbiology.net/gtc

**Operating system: **Platform independent web site

**Programming languages: **Java, Javascript

**Other requirements: **Java 1.5 or higher and a web browser. While GTC can be viewed with any modern web browser, Firefox and the Firegoose extension are required for full interoperability with Gaggle-enabled applications and websites.

http://getfirefox.com

http://gaggle.systemsbiology.net/docs/geese/firegoose/

**License: **GNU LGPL

**Any restrictions to use by non-academics: **None

## Competing interests

The authors declare that they have no competing interests.

## Authors' contributions

DT developed the specification and design of the software and drafted the manuscript. JCB contributed to the design of the software. NSB conceived of the project, participated in its design and coordination and helped to draft the manuscript. All authors read and approved the final manuscript.
